# Determinants of Latine Patients’ Trust in an Immigrant-Inclusive Health Care Institution: A Mixed-Methods Study

**DOI:** 10.1177/24731242261421854

**Published:** 2026-03-10

**Authors:** Altaf Saadi, Margarita G. Velasco, Christine Tzelios, Stephanie Lemus, Krystal E. Kuan, Tessa Wilson, Michele Heisler

**Affiliations:** 1 Harvard Medical School, Boston, Massachusetts, USA.; 2 Department of Neurology, Massachusetts General Hospital, Boston, Massachusetts, USA.; 3 Department of Medicine, Massachusetts General Hospital, Boston, Massachusetts, USA.; 4 Department of Central American and Transborder Studies, California State University, Northridge, Los Angeles, California, USA.; 5 Department of Epidemiology, Boston University School of Public Health, Boston, Massachusetts, USA.; 6 Physicians for Human Rights, New York, New York, USA.; 7 Department of Internal Medicine, School of Public Health, University of Michigan, Ann Arbor, Michigan, USA.

**Keywords:** immigrant health, medical mistrust, institutional trust, health disparities

## Abstract

**Introduction:**

Past research has overlooked both the institutional factors of medical mistrust and immigrants’ perspectives. We aimed to understand factors influencing Latine immigrant patients’ medical mistrust, focusing on their perspectives of immigrant-inclusive institutional policies.

**Methods:**

We conducted a sequential explanatory mixed-method study at a Federally Qualified Health Center (FQHC) in Los Angeles, CA, involving surveys (*N* = 170) and interviews (*n* = 20). Survey and interview data were descriptively and thematically analyzed, respectively.

**Results:**

The mean age of the total sample was 54.5 years, among whom 69.4% were women. Patients trusted physicians more than health care systems. Group-based medical distrust related to immigrant identity was below the median, though participants highlighted differences in their more “immigrant-friendly” local context. Over half expressed moderate-to-high worry about deportation, with nearly a quarter avoiding health-related activities due to these fears. However, 99% still visited their health care center and most (92.9%) had received the COVID-19 vaccination. Among “immigrant-friendly” institutional policies, confidentiality of patient information was ranked most important. The interviews revealed six themes on the determinants of trust: (1) confidentiality, (2) in-person clinical encounters, (3) perceptions in social networks, (4) prior health care experiences, (5) health system staff and processes, and (6) addressing social needs.

**Conclusion:**

Medical trust is shaped by interconnected micro- and macrolevel relationships, requiring multilevel efforts to strengthen it. For Latine immigrants, this includes institutional immigrant-inclusive policies, efforts to address social needs, patient-centered and diverse staff, and efficient processes of care.

## Introduction

Medical mistrust—the belief that health care-affiliated individuals or institutions work against one’s best interests^1^—is a significant barrier to health, contributing to reduced cancer screening, medication adherence, vaccination, quality of life, and patient satisfaction.^2–4^ Operating at both interpersonal and institutional levels, medical mistrust requires strategies that address both to effectively improve health outcomes and patient engagement.^1^ However, prior research has largely focused on interpersonal trust (i.e., improving patient–provider communication^5^), even though patients’ trust of individual providers is not consistently correlated with trust of health care systems.^6^ This highlights the need to address institutional trust^7^—defined here as patients’ relationships with various elements of the health care system, including hospitals, insurers, pharmaceutical companies, and medical research.^1^ Improving institutional trust means shifting focus from interpersonal interactions to making health care institutions more trustworthy.^8^

Institutional mistrust is rooted in a legacy of racism and exploitation in health care and disproportionately affects marginalized groups like Black and Latine patients, who report greater medical mistrust than White patients.^9^ For Latine patients, historical exploitation includes the Guatemala Syphilis experiments^10^—where doctors/researchers intentionally infected thousands with sexually transmitted disease without treatment—and forced sterilization of hundreds of Latine individuals in California.^11,12^

Immigrants, particularly those without stable immigration status, face distinct challenges that make addressing medical mistrust even more urgent. They navigate unique stressors such as immigration enforcement, restrictive immigration policies, and anti-immigrant rhetoric—all of which deter health care use and portend poorer health outcomes.^13^ These harms extend to immigrants with authorized immigration status, U.S. citizens in mixed immigration-status households, and those of the same ethnic communities due to racialized assumptions about “legality.”^13^

Over the last several decades, exclusionary immigration policies and rhetoric have eroded medical trust among immigrants, leading to reduced health care use,^14^ lower confidence in the government as a source of health information,^15^ and lower participation in public programs.^16^ The COVID-19 pandemic marked another significant turning point in immigrants’ experiences of medical mistrust. Immigrants were disproportionately exposed to occupational hazards and faced higher risks of infection, illness, and death, as well as greater rates of food and housing insecurity and unemployment—all of which deepened existing health disparities.^17^ At the same time, many immigrants faced fears about public charge^17^—fearing that seeking health care might jeopardize their or their family’s future legal residency—alongside concerns about information sharing between public health agencies and law enforcement.^18^ Consequently, immigrants had decreased vaccine uptake,^19,20^ despite their heightened vulnerability to infection and mortality. These fears, which had already intensified after the 2016 U.S. presidential election,^21,22^ have further deepened under the second Trump administration’s renewed exclusionary immigration policies,^23^ including rescission of prior guidance limiting immigration enforcement in health care facilities.^24^ Understanding immigrants’ perspectives on health care institutions’ trustworthiness is therefore critical.

Prior research has identified health care institution policies and actions to create immigrant-inclusive institutions—such as limiting immigration status documentation in medical records, preparing for immigration enforcement on health care facility premises, addressing legal concerns and promoting awareness of legal rights, and creating virtual hubs for credible sources of health information.^8,25–28^ Yet little is known about how patients perceive these efforts, and such perspectives are largely absent from medical mistrust literature. This study addresses these gaps by examining how predominantly foreign-born Latine patients at an immigrant-inclusive clinic perceived these institutional policies and determinants of trust. We also explored how the COVID-19 pandemic shaped their immigration-related fears, experiences, and health care behaviors.

## Methods

### Study design and setting

This sequential explanatory mixed-method study comprised a sample of adult patients from a Federally Qualified Health Center (FQHC) predominantly serving low-income, immigrant Latine individuals in Los Angeles, CA. This FQHC was chosen as it had implemented a range of immigrant-inclusive institutional policies and practices.^25^ The study was deemed exempt by the Massachusetts General Hospital Institutional Review Board.

### Data collection

Participants were (1) 18 years or older, (2) Spanish or English speakers, (3) foreign-born immigrants or second-generation immigrants (i.e., they had a parent who had immigrated), and (4) had at least one visit to the clinic in the prior year. There was no capacity to conduct surveys or interviews in other languages. *Promotores de salud*, or Community Health Workers (hereafter, *promotores*), referred participants from their routine patient interactions, consistent with prior research showing them to be effective in recruiting hard-to-reach participants.^29^ Research team members then contacted potential participants to provide study information and obtain informed consent. Surveys were conducted telephonically from August 2021 to February 2022.

Following preliminary analysis of survey data, a purposive sample of participants using maximum variation sampling was selected for semistructured interviews.^30^ Namely, we contacted individuals who had consented to be contacted for an interview and represented the highest and lowest levels of trust on these scales. We continued conducting interviews across this range of trust levels until we reached data saturation (e.g., no new themes emerged during our ongoing analysis). Interviews (approximately 60 min in duration) were conducted telephonically by one bilingual team member, who herself is a Latina immigrant, from December 2021 to April 2022, and professionally transcribed and translated. All participants received a $10 gift card for the survey and a $10 gift card for the interview. To ensure confidentiality, verbal consent was obtained, no personal identifiers were recorded, transcripts were de-identified for analysis, and recordings were destroyed after the transcription was reviewed for accuracy.

#### Quantitative survey

In addition to demographic questions, the survey assessed knowledge of and opinions on immigrant-inclusive policies at their health center, as identified in prior research.^25^ We included three validated trust measures: (1) the 11-item Trust in Physicians Scale,^31,32^ with responses on a 5-point Likert scale (1 “strongly disagree” to 5 “strongly agree”) transformed to a 100-point scale (higher scores reflect greater trust); (2) the 12-item Group-based Medical Distrust Scale, assessing immigrant group distrust in the health care system,^33^ similarly transformed to a 100-point scale for consistency and interpretation across scales (higher scores indicate more distrust); and (3) the 10-item Health Care System Distrust Scale,^6^ also using a 5-point Likert format transformed to a 100-point scale (higher scores indicate more distrust). The survey also included items on deportation fears (used in previous studies^34^), the use of health services, and changes in attitudes since the COVID-19 pandemic.

#### Qualitative interviews

Interview questions built on survey topics, exploring factors influencing trust in health care institutions; ways to strengthen that trust; policies or programs to make health centers more welcoming to immigrants; and how the COVID-19 pandemic may have affected these determinants (Supplementary Fig. S1). The questions were developed independently from the quantitative data. We included exploratory questions relating to perceptions of law enforcement in health care settings and trust in biomedical research, reported elsewhere.^35,36^

### Data analysis

Survey data were analyzed using descriptive statistics, using SAS software (version 9.4). We used thematic analysis to analyze qualitative data.^37^ Two study team members independently reviewed each de-identified transcript and compared codes, which were later grouped into themes. Disagreements were resolved through consensus and, if needed, by a third study team member. The preliminary codebook was revised iteratively. We used Dedoose (version 9.0.54) for qualitative data management. Preliminary results were presented to clinic staff and *promotores* to solicit impressions about the results. There was agreement that the data collected were consistent with their perceptions.

## Results

### Sample description

A total of 170 people completed the survey, and 20 completed the interviews (see Fig. 1). Participant characteristics are presented in Table 1. The mean age was 54.5 years, and 69.4% were women. All were immigrants from Central America or Mexico, except for three participants whose parents had immigrated. Participants had lived in the United States for an average of 24.8 years. A majority (58.6%) had less than an eighth-grade education. Most (76.5%) reported having a primary care physician, with 68.5% reporting having “poor” or “fair health.”

**FIG. 1.**
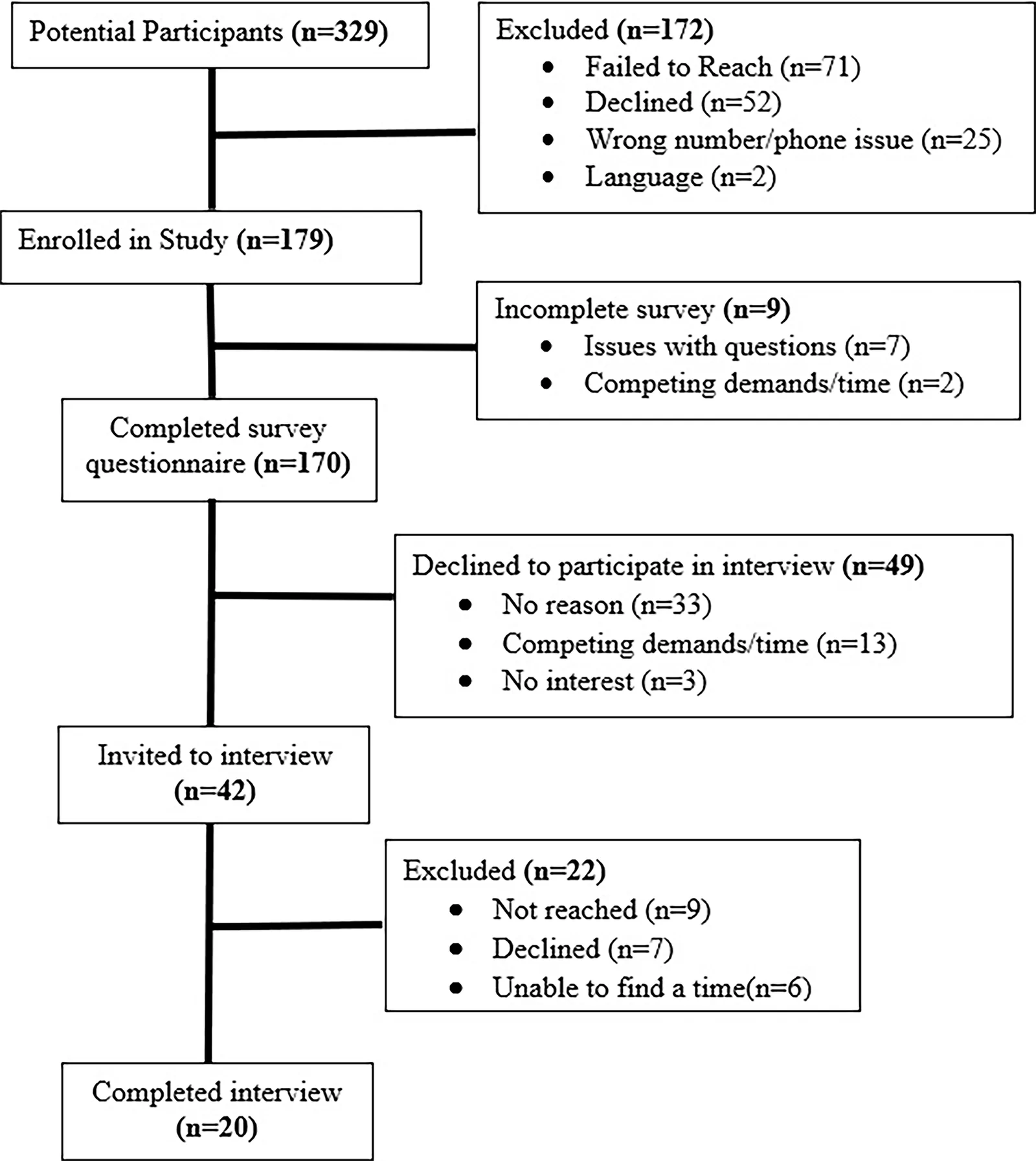
Study flow diagram.

**Table 1.** Participant Characteristics (*N* = 170)

Characteristic	
Age, mean (SD) [range]	54.5 (11.4) [19–84]
Female, *n* (%)	118 (69.4%)
Education, *n* (%)	
<8th grade	99 (58.6%)
8th grade to ≤HS	54 (32%)
Some college and above	16 (9.5%)
Hispanic/Latino ethnicity	170 (100%)
Country of origin (169)	
Mexico	77 (45.3%)
El Salvador	49 (28.8%)
Guatemala	29 (17.1%)
Honduras	11 (6.5%)
United States	3 (1.8%)
Language spoken at home, *n* (%)	
Spanish	163 (95.9%)
English	1 (0.6%)
Other (indigenous languages)	6 (3.5%)
English proficiency, *n* (%)	
Well/Very well	147 (89.1%)
Not well/Not at all	18 (10.9%)
Years lived in the United States, mean (SD) [range]	24.8 (9.6) [0.3–51]
Self-reported health, *n* (%)	
Excellent/Very good/Good	53 (31.6%)
Fair/Poor	115 (68.5%)
Have a regular primary care physician, *n* (%)	130 (76.5%)

SD, standard deviation.

### Trust measures and immigration-related fears

Table 2 presents participants’ mean scores on physician and health system trust levels. Trust in physicians was high, with an average score of 62.0 out of 100 (higher scores indicating greater trust). Health care system distrust was at the median, with an average score of 50.5 out of 100 (higher scores indicating greater distrust). Group-based medical distrust scale, meaning distrust based on one’s group identity (in this study, immigrant identity), was lower than the median, with an average score of 43.9 out of 100 (higher scores indicating greater distrust).

**Table 2.** Participant Responses on Measures of Trust and Deportation Fears

Variable	
Physician Trust Scale, mean (SD) (*n* = 115)	62.0 (8.5)^a^
Group-Based Medical Distrust Scale (distrust based on one’s group identity), mean (SD) (*n* = 99)	43.9 (9.7)^b^
Health Care System Distrust Scale, mean (SD) (*n* = 107)	50.5 (10.0)^b^
Fear of deportation, *n* (%) (*n* = 144)	
Not at all worried	32 (22.2%)
A little worried	36 (25.0%)
Moderately worried or higher	76 (52.8%)
Avoidance of health-related activities for fear of deportation, *n* (%) (*n* = 170)	38 (22.4%)
Visits to (vs. driving to) health care facilities	2 (1.2%)
Scheduling health care appointments	4 (2.4%)
Enrolling in health insurance (vs. Medicaid)	8 (4.7%)
Enrolling in SNAP (Supplemental Nutrition Assistance Program, i.e., for food stamps)	36 (21.2%)
Enrolling in WIC (Women, Infants, and Children)	20 (11.8%)

a On a 100-point scale, higher scores represent higher trust.

b On a 100-point scale, higher scores represent higher distrust.

Participants reported that their concerns about immigration status influenced their health care behaviors: 52.8% were moderately or very worried about deportation, with 21.2% avoiding health-related activities due to this fear (Table 2).

### Awareness and perspectives on immigrant-inclusive institutional policies

Table 3 summarizes participants’ awareness and perceived importance of various immigrant-inclusive institutional policies. Overall, participants considered all listed policies as high priority (all means of 9 or greater out of 10). Most (83.5%) were aware of policies to keep patient information confidential, ranking its importance very high with a mean of 9.5 (SD 1.5). Over half knew about policies regarding educating patients about their legal rights (61.8%) and the nonquestioning of immigration status by clinicians and staff (57.7%). Both policies were rated similarly, with means around 9.5 and 9.0, respectively. Although awareness of other policies was lower, they were still perceived as highly important.

**Table 3.** Awareness and Ranked Importance of Institutional Policies to Create Immigrant-Friendly Climate

Policy	Yes, *n* (%)	Ranked importance^a^ (1–10), mean (SD)
Protecting patient information and keeping patient information confidential	142 (83.5%)	9.5 (1.5)
Educating patients about their legal rights and privacy rights	105 (61.8%)	9.5 (1.5)
Clinicians and staff not asking about immigration status	98 (57.7%)	9 (2.1)
Physical signs or other messages that welcome immigrant patients	91 (53.5%)	9.2 (1.8)
Clinicians and staff not documenting immigration status in medical records	70 (41.2%)	9.1 (2.1)
Clinicians and staff helping patients prepare for emergency situations like if they get detained or deported	65 (38.2%)	9.5 (1.5)
Signs that indicate what is a public or private space so that immigration officers cannot enter private patient areas without a warrant	56 (32.9%)	9 (2.2)
Partnering with local legal organizations or referring to legal services if a patient needs it	51 (30%)	9.2 (1.8)
Limiting cooperation with immigration enforcement	45 (26.5%)	8.7 (2.3)
Other	20 (11.8%)	NA

a 1 being not important and 10 being the most important.

### Pandemic-specific immigration-related fears and experiences

Table 4 summarizes participants’ responses to questions about the effect of COVID-19 on their immigration-related fears and experiences. About one in five participants reported heightened fears of immigration enforcement overall (22.4%), including specifically at or near health care facilities (24.1%), and increased fears about their immigration status being shared with government officials (20.6%). A similar proportion (22.4%) reported experiencing discrimination or bullying regarding their race, ethnicity, or immigration status. Despite these challenges, a high percentage (92.9%, *n* = 158) received the COVID-19 vaccine.

**Table 4.** COVID-19 Pandemic Immigration-Related Fears and Experiences

Experience^a^	Mean (^b^Likert scale from 1 to 5)	SD
Policies at my health care facilities have changed to better protect immigrants.	3.5	0.9
Disagree/Strongly disagree	28 (16.5%)	
Neutral	17 (10.0%)	
Agree/Strongly agree	96 (56.5%)	
Missing	29 (17.1%)	
My health care use (how often I use health care services) has decreased.	3	1
Disagree/Strongly disagree	79 (46.5%)	
Neutral	10 (5.9%)	
Agree/Strongly agree	77 (45.3%)	
Missing	4 (2.4%)	
My health care access (having health insurance, having access to specialists, and being able to attend appointments) has decreased.	3	1
Disagree/Strongly disagree	80 (47.1%)	
Neutral	10 (5.9%)	
Agree/Strongly agree	75 (44.1%)	
Missing	5 (2.9%)	
My fear of immigration enforcement has increased.	2.8	1
Disagree/Strongly disagree	78 (45.9%)	
Neutral	19 (11.2%)	
Agree/Strongly agree	38 (22.4%)	
Missing	35 (20.6%)	
My fear of immigration enforcement at or near health care facilities or COVID-19 testing sites has increased.	2.6	0.9
Disagree/Strongly disagree	102 (60.0%)	
Neutral	11 (6.5%)	
Agree/Strongly agree	41 (24.1%)	
Missing	16 (9.4%)	
I have experienced increased levels of discrimination or bullying regarding my race, ethnicity, or immigration status.	2.5	0.9
Disagree/Strongly disagree	117 (68.8%)	
Neutral	5 (2.9%)	
Agree/Strongly agree	38 (22.4%)	
Missing	10 (5.9%)	
My fear of my immigration status being provided to government officials has increased.	2.5	0.9
Disagree/Strongly disagree	111 (65.3%)	
Neutral	9 (5.3%)	
Agree/Strongly agree	35 (20.6 %)	
Missing	15 (8.8%)	
I have increased symptoms of anxiety or depression due to fear of immigration enforcement.	2.5	0.9
Disagree/Strongly disagree	127 (74.7%)	
Neutral	8 (4.7%)	
Agree/Strongly agree	32 (18.8%)	
Missing	3 (1.8%)	
My fear of driving to my health care facility has increased.	2.4	0.8
Disagree/Strongly disagree	128 (75.3%)	
Neutral	6 (3.5%)	
Agree/Strongly agree	28 (16.5%)	
Missing	8 (4.7%)	
I have experienced increased levels of discrimination or bullying from my health care provider and/or other health care workers regarding my race, ethnicity, or immigration status.	2.3	0.7
Disagree/Strongly disagree	134 (78.8%)	
Neutral	12 (7.1%)	
Agree/Strongly agree	18 (10.6%)	
Missing	6 (3.5%)	

a Distributions are reported as *n* (%), unless otherwise specified.

b Responses were on a Likert scale from 1 “strongly disagree” to 5 “strongly agree.” Higher scores indicate more agreement with the statement.

### Qualitative results from semistructured interviews

We identified six themes on the determinants of trust in the health care system: (1) confidentiality; (2) in-person clinical encounters; (3) perceptions in social networks; (4) prior health care experiences; (5) health system staff and processes; and (6) addressing social needs. Participants did note that trust in the health care system overall mattered less during emergencies, as patients had less agency in decision-making and considering institutional trustworthiness.

1) *Confidentiality*

Most participants mentioned the importance of patient information privacy and confidentiality in generating trust. As a 68-year-old man from El Salvador said: *“So that the patient can trust the place… there should be privacy.”*

This included not being asked about their immigration status, as in the words of this 74-year-old man from El Salvador:

They [the hospitals] don’t ask you if you have legal documents or not. If you go there sick, they always treat you. Some hospitals are neutral, they don’t care if you have documents or not, everyone is worth the same.

This prioritization of confidentiality as a determinant of trust was contextualized within fears about immigration status disclosure and potential risk of deportation. However, many repeated that these concerns were reduced in Los Angeles city that was perceived as more immigrant-friendly.

2) *In-person clinical encounters*

Some participants reported that virtual visits during the pandemic reduced their trust in health care, with access and technical issues common. As one 51-year-old woman from Mexico explained:

[O]ver the phone is not the same as talking to the doctor in person… This is the distrust one has. Since you don’t know if the doctor is really paying attention to you or whether he is ignoring you… I feel more comfortable talking face to face with the doctor, you understand?

3) *Perceptions in social networks*

Most participants mentioned that their social networks (i.e., family and friends) helped build their trust in institutions. As one 44-year-old man from El Salvador said:

Because of what I heard before from people who have gone to some healthcare center or to some hospital, about the care they have received, that is one of the ways that one can trust.

4) *Prior health care experiences*

Most participants’ trust was shaped by past health care experiences in both the United States and their home countries. In the United States, trust increased with positive prior clinical experiences and declined with perceived ineffectiveness. Further, while they acknowledged the U.S. system’s greater resources than their home countries, many perceived it as costly and driven by profit motives, which undermined trust. As a 62-year-old woman from El Salvador said:

There are times when medicines that are not so expensive… there are clinics that inflate the prices… they make them more expensive… I have knowledge of that, because I am from El Salvador and there are medicines that I buy there…and here they triple and quadruple [the cost].

5) *Health system staff and processes*

Some participants described health care system workflow factors, such as waiting times, prompt appointments, and communication between various components of a health care organization (e.g., specialty referrals, pharmacy) as influencing their trust. As one 74-year-old man from El Salvador said:

Let me tell it to you straight… every time I make an appointment, they tell me they’ll call me back and I wait, and I wait, and they never call me. That’s why I don’t trust them, you see.

Clinician turnover was also mentioned in this context, as repeated changes required rebuilding relationships *“little by little*.” Conversely, long-term presence—both individual and institutional—fostered trust. For example, a 61-year-old woman from El Salvador described trusting a long-established “*recognized clinic*” more than *“street clinics… that they put them in and take them out*.*”*

Individual relationships with staff were cultivated through a perceived culture of respect for immigrants and diverse health care staff. Staff included people at the front desk to physicians; in fact, the majority discussed attentive and caring nonclinical staff explicitly, like this 73-year-old man from El Salvador:

When I arrive, there is someone who gives me what I need. You need this? Then come here or wait here. They give me a good explanation… And if they don’t have, maybe… they don’t speak Spanish, they find someone who speaks Spanish, and they tell me what I need to do.

Finally, a few participants mentioned health care facility’s physical infrastructure as influencing their trust, for example, having chairs in the waiting room, and the cleanliness of the facility.

6) *Addressing social needs*

Most participants described how health care systems’ addressing social factors like discrimination, cost, and health literacy increased their trust in the health care system; it made them feel valued as whole individuals, not just *“a number.”* Conversely, experiences of discrimination in health care spaces, often on multiple intersecting fronts—being an immigrant, *“because they’re poor,”* having public rather than private insurance, speaking Spanish or a language other than English—decreased their trust. As a 68-year-old man from El Salvador explained:

[The clinic] they treat just anyone… I mean people go there, it doesn’t matter their immigration status, they offer what they can… and you get the attention you need, and they make you feel that you are at a place you can trust… You know that you will not be rejected because you can’t pay.

Health care systems further cultivated trust by providing robust language services through both interpreters and bilingual and culturally concordant staff. As a 39-year-old woman from Mexico explained: *“When I went for prenatal care for my first pregnancy, I felt way better… because the nurses were Latinas, they spoke Spanish, they explained everything.”*

Health care systems also cultivated trust by providing information about immigration-related issues. As a 48-year-old man from El Salvador explained: *“To touch on some issues that a person, like an immigrant to this country, doesn’t know, like immigration issues… like with flyers, a place to go for immigration problems.”*

## Discussion

Among this sample of largely foreign-born Latine immigrants who receive care at an immigrant-inclusive FQHC, trust in physicians was high, while their trust in health care systems was lower. This reflects prior findings of a mismatch between physician and health care system trust,^34,38^ highlighting the need to consider medical mistrust as multifaceted, targeted at both interpersonal and institutional levels. Notably, participants in our study emphasized that trust extended beyond clinicians to all clinic staff, highlighting why we must envision trust as a web—a concept often overlooked in research focused solely on clinicians as influencing the patient experience.^39^

More than half of participants reported being moderately or very worried about deportation, and almost a quarter of participants reported avoiding health-related activities due to the fear of deportation (i.e., enrolling in programs such as SNAP or WIC); yet 99% reported that they did not avoid visiting their health care center due to fear of deportation. Reassuringly, despite misinformation during the COVID-19 pandemic, and a national decline in trust of physicians and health care systems,^40^ >95% of participants reported receiving a COVID-19 vaccination, higher than reported in national samples of Latine Americans and immigrants.^41^

Institutional policies to promote an immigrant-inclusive health system may represent one example of a trust-facilitating factor that promotes positive health behaviors. In this study, there were high rates of awareness (83.5%) among participants that the clinic would keep all their patient confidential, something perceived as greatly important in survey ratings and interviews. Almost 60% of participants were also aware that no clinic staff would ask about their immigration status. All the immigrant-friendly policies instituted at the FQHC were rated as highly important, though lower rates were aware that these policies had been enacted. This reinforces the importance of both instituting policies and publicizing them among patients to increase awareness of them. In a multistate study assessing the early implementation of such institutional policies, most health care systems were FQHCs.^25^ However, broader adoption is needed, especially given the recent reversal of prior guidance that had restricted immigration enforcement in health care facilities.^24^

Participants also highlighted other health care system efforts as essential for fostering trust. This included addressing patients’ social needs, staff patient-centeredness, and ensuring cultural and linguistic diversity. Previous research has linked addressing social needs to better patient health outcomes and institutional financial returns on investment.^42^ Our study offers that one mechanism behind these positive health outcomes may be through improved institutional trustworthiness and therefore positive engagement with the health care system. Staff training and increased workforce diversity may also help reduce experiences of discrimination in health care settings, which can further enhance positive engagement with health care. The detrimental impact of discrimination on positive engagement with health care systems has been emphasized in prior research.^43^ Furthermore, participants in our study suggested that even physical space factors like chairs, clutter, maintenance, staff turnover, and health system fragmentation influenced trust, underscoring the need to expand our current understanding of trust beyond the interpersonal and to consider multilevel interventions such as institutional policies, physical environment, and changes to local and state policies.^44^

At the interpersonal level, participants preferred in-person over virtual visits, underscoring the importance of personal relationships in building trust. Future efforts should consider trust-building in determining the balance between virtual and in-person visits. While telemedicine raises concerns about digital access and privacy,^18^ it also offers advantages that must be carefully weighed (e.g., reducing travel time, mitigating fears relating to immigration enforcement when driving to a clinic). Participants also emphasized social networks as key to promoting trust, aligning with prior research on the role of health care-community partnerships.^44^

Group-based medical distrust scale, meaning distrust based on one’s group identity (in this study, immigrant identity), was lower than the median. However, participants noted differences in the local context of Los Angeles, CA, as a more immigrant-inclusive city. Prior research has identified varying levels of inclusive versus exclusionary policies at local, state, and federal levels that influence health use and outcomes.^45–47^ For example, a small percentage of our cohort identified fear of driving to health care facilities. This fear is likely mitigated by state policy allowing for driver’s licenses for all residents, irrespective of immigration status. This fear may be more salient in states where similar policies do not exist. It is likely that group-based medical mistrust varies according to local, state, and federal contexts.

## Limitations

Our study has its limitations. First, this was a convenience referral sample from *promotores*, with these trusted intermediaries potentially recruiting participants who are more trusting. We observed a higher proportion of older Latine adults, primarily of Mexican heritage, with longer tenure in the United States, which may contribute to bias. For example, greater acculturation may be associated with greater trust.^48^ Future research should include more diverse Latine samples, particularly recent arrivals, to better capture variations within the Latine population. Second, our study represents only one FQHC and one health care institution type. There may be differences across FQHCs and health care institution types like academic medical centers or county facilities. Relatedly, it will be important to replicate our study in clinics in cities and states with higher levels of immigration enforcement and/or perceived as less immigrant-friendly than Los Angeles, California.

## Conclusion

This study is the first to evaluate trust levels and determinants among Latine immigrants receiving care at a clinic with immigrant-inclusive institutional policies, bridging gaps in both the medical mistrust and immigrant-inclusive policy literature. Further, while some U.S. clinics have adopted such policies,^14^ this is the first to explore patients’ awareness of and perspectives on these policies. Findings highlight the importance of institutional immigrant-inclusive policies among patients, alongside efforts to address social drivers of health and ensure respectful, efficient care by a diverse workforce. We also emphasize the need to view trust as “determined a ‘web’ of mutually interacting relationships between individuals and social systems.”^49^ Understanding trust as a web of mutually interacting micro- and macrolevel relationships helps to inform multilevel interventions. It could also explain why interventions focused on interpersonal trust have fallen short.^50^ This multilevel understanding is especially critical amid evolving and increasingly exclusionary local, state, and federal immigration policies.

## Authors’ Contributions

A.S. contributed to study conceptualization, methodology, data analysis, interpretation, provision of resources, writing, and study supervision. M.V. contributed to data curation, data analysis, interpretation, writing, and project administration. C.T. contributed to data curation, interpretation, and writing. S.L. contributed to data curation and writing. K.E.K. contributed to data analysis, interpretation, and writing. T.W. contributed to writing. M.H. contributed to study methodology, data interpretation, and writing.

## Data Availability

The data that support the findings of this study are available on request from the corresponding author, Altaf Saadi. The data are not publicly available due to containing information that could compromise the privacy of research participants.

## Supplemental Material

sj-tif-1-heq-10.1177_24731242261421854 — Supplemental material for Determinants of Latine Patients’ Trust in an Immigrant-Inclusive Health Care Institution: A Mixed-Methods Study Supplemental material, sj-tif-1-heq-10.1177_24731242261421854 for Determinants of Latine Patients’ Trust in an Immigrant-Inclusive Health Care Institution: A Mixed-Methods Study by

## Abbreviations

CA: California
FQHC: Federally Qualified Health Center
SNAP: Supplemental Nutrition Assistance Program
WIC: Women, Infants, and Children
